# Antioxidant Effect of Ethyl Acetate Fraction from *Kaempferia galanga* L.: Integrated Phytochemical Profiling, Network Analysis, and Experimental Validation

**DOI:** 10.3390/antiox14050551

**Published:** 2025-05-05

**Authors:** Siyu Wang, Jianzhan Yang, Lei Cai, Haoxiang Li, Xiaodong Han, Bo Liu, Jianwei Wu

**Affiliations:** 1 School of Traditional Chinese Medicine and Health, Nanfang College Guangzhou, Guangzhou 510970, China; wangsy@nfu.edu.cn; 2 Chinese Medicine Guangdong Laboratory, Zhuhai 519060, China; 20221110782@stu.gzucm.edu.cn (J.Y.); 20222110162@stu.gzucm.edu.cn (H.L.); 3 Guangdong Provincial Key Laboratory of Clinical Research on Traditional Chinese Medicine Syndrome, The Second Clinical Medical College, Guangzhou University of Chinese Medicine, Guangzhou 510006, China; ygb@gzucm.edu.cn; 4 Guangdong Provincial Biotechnology Research Institute (Guangdong Provincial Laboratory Animals Monitoring Center), Guangzhou 510663, China; cail@gdlami.com; 5 Guangzhou Key Laboratory of Chirality Research on Active Components of Traditional Chinese Medicine, Guangzhou 510006, China; 6 State Key Laboratory of Dampness Syndrome of Chinese Medicine, Guangzhou 510006, China

**Keywords:** *Kaempferia galanga* L., antioxidant activity, network pharmacology, molecular docking, molecular dynamics simulation, RAW264.7 cells, PI3K/Akt and MAPK signaling pathways, zebrafish

## Abstract

*Kaempferia galanga* L. is well known for its use in medicinal and edible homologous application. Various diseases, including those related to oxidation, are commonly treated with it. However, its antioxidant effect is still lacking systematical study. We aimed to screen the most potential antioxidant fraction of the crude ethanolic extract from *K. galanga* (KG) and evaluate its antioxidant activity and potential mechanism. The ethyl acetate fraction of ethanolic extract from *K. galanga* (KGEA) was chosen as the most potent antioxidant activity from all the fractions tested. UPLC-Q-TOF-MS/MS was used to determine 43 compounds in KGEA, and 25 potential bioactive compounds were identified by pharmacokinetic analysis. Network pharmacology revealed 174 overlapping targets of chemical and antioxidant targets, and the key targets were identified. Molecular docking and MD simulation revealed a strong binding affinity between the core compounds and their targets. In tests against DPPH and ABTS, KGEA exhibited potent radical scavenging activity. In H_2_O_2_-induced cells, KGEA could decrease reactive oxygen species (ROS) production; alleviate mitochondrial damage; promote the increase in antioxidant enzymes SOD, CAT, GSH-Px; and reduce the levels of MDA. Mechanistically, KGEA regulated PI3K/Akt and MAPK signaling pathways against oxidative damage. Moreover, in H_2_O_2_-induced zebrafish, KGEA attenuated ROS generation, cell death, lipid peroxidation, and increased SOD, CAT, GSH-Px activities; it also decreased MDA levels. The antioxidant properties of KGEA were demonstrated in vitro and in vivo, and it should be considered as an antioxidant agent for further profound study.

## 1. Introduction

When organisms are exposed to reactive nitrogen or oxygen radicals beyond their scavenging limits, the organisms are in an imbalanced state of oxidative stress [1]. Many environmental factors, such as ultraviolet (UV), ionizing radiation, and chemotherapeutics produce reactive oxygen species (ROS), cause oxidative stress damage to the cells [2]. Various diseases, including cancer, diabetes, autoimmune diseases, and cardiovascular diseases, are thought to be related to ROS imbalance [3,4,5]. Therefore, the balance between ROS production and antioxidant defense is significant in maintaining the normal physiological function of organisms. The antioxidants help to prevent against diseases via regulating cell growth, apoptosis, and differentiation pathways [6]. A growing body of research indicates that antioxidants are biologically essential in combating ROS and food is an important source of antioxidant supplements [7]. Therefore, studies on antioxidants in food, especially in plants, are of great interest [8].

For thousands of years, Traditional Chinese Medicine (TCM) has been used to prevent and treat diseases with its multi-ingredient, multitarget properties [9]. With the deepening of modern pharmacological understanding of TCM mechanism, TCM medications have increasingly been used as antioxidants.

*K. galanga*, a species within the *Kaempferia* L. genus of the Zingiberaceae family, is a well-regarded medicinal herb extensively utilized in various regions such as India, China, Myanmar, Bangladesh, and Thailand [10]. In folk medicine, the roots are used for the treatment of toothaches, asthma, rheumatism, and wounds [11]. Additionally, *K. galanga* is widely used as a flavoring agent, thereby serving dual purposes as both a medicinal and culinary resource. Pharmacological studies demonstrate that *K. galanga* possesses antioxidant, antimicrobial, anti-inflammatory, and antitumor properties [12,13,14,15]. Methanolic extracts of *K. galanga* have shown antioxidant activity, as assessed by DPPH and ABTS scavenging assays [15]. Furthermore, DPPH radical scavenging tests indicate that the leaves of *K. galanga* exhibit limited antioxidant activity [16]. Notably, the antioxidant activity of *K. galanga* ethanol extract has not been systematically investigated, leaving its effective fractions, potential antioxidant components, and associated signaling pathways still unclear. Given the preference for natural antioxidants derived from medicinal plants over synthetic alternatives, it is imperative to conduct comprehensive studies on the antioxidant activity of *K. galanga* fractions, as well as to elucidate its chemical constituents and the underlying mechanisms involved.

In view of the complex active compounds in *K. galanga* and the pharmacological mechanisms connected to several signaling pathways, it is difficult to determine the mechanism of antioxidation. Due to the multicomponent and multitarget characteristics of herbal medicines, network pharmacology has emerged as a robust research methodology. Network pharmacology was proposed by Hopkins in 2007, which is the theory based on systems biology [17]. It includes multi-disciplinary technologies and is widely used as a research approach that could construct a multi-level network of drug–target–disease to display the relationship between drugs and diseases and explore the underling mechanism of drugs [18].

In network pharmacology, basic processes include the collection of component targets, the acquisition of disease targets, enrichment analysis, and the verification of key targets [9,19]. Network pharmacology offers valuable insights into the molecular mechanisms of TCM in addressing various diseases and remains a promising approach for discovering natural therapeutics. For examining the docking modes between small molecules and proteins, molecular docking has been widely applied in computer-aided drug design [20]. To evaluate the stability and adaptability of the binding between active compounds and therapeutic targets, molecular dynamics (MD) simulation was employed on ligand–receptor complexes. Identifying compounds with potentially therapeutic effects on diseases has been successfully achieved in TCM using these techniques [21,22]. However, a systematic understanding on multiple therapeutic targets exerting antioxidant effects of *K. galanga* needs to be further developed.

In the present study, we aimed to screen the best antioxidant activity fraction of *K. galanga* and identify its components using UHPLC-QE-MS analysis. Then, network pharmacology, molecular docking, and MD simulation were conducted to explore its potential molecular mechanisms. Meanwhile, hydrogen peroxide (H_2_O_2_)-induced RAW 264.7 cells and a H_2_O_2_-induced zebrafish model were applied to evaluate its antioxidative effects.

## 2. Materials and Methods

### 2.1. Materials

Acetonitrile and formic acid were purchased from Merck & Co., Inc. (Rahway, NJ, USA). 2,2-diphenyl-1-picrylhydrazyl (DPPH; 99.13%), 2,2′-amino-di(2-ethyl benzothiazoline sulfonic acid-6) ammonium salt (ABTS; 99.86%), ascorbic acid (≥99%), were obtained from MedChemExpress LLC (Monmouth Junction, NJ, USA). All cell reagents were purchased from Life Technologies Co. (Carlsbad, CA, USA). Hydrogen peroxide (H_2_O_2_), 2′,7′-dichlorodihydrofluorescein diacetate (DCF-DA) and acridine orange were acquired from Sigma-Aldrich Co. (St. Louis, MO, USA). Diphenyl-1-pyrenylphosphine (DPPP) was obtained from Shanghai Macklin Biochemical Co., Ltd. (Shanghai, China). Malondialdehyde (MDA), superoxide dismutase (SOD), catalase (CAT), and glutathione peroxidase (GSH-Px) test kits were obtained from Nanjing Jiancheng Bioengineering Institute (Nanjing, China).

### 2.2. Plant Material and Discovery of Bioactive Fraction in K. galanga

*K. galanga* rhizomes were obtained from the Qingping market for Chinese medicinal herbs in Guangdong Province, China, and validated by Junbiao Wu, the deputy chief pharmacist from Guangzhou University of Chinese Medicine in Guangzhou, China. The dried samples (1 kg) were ground into powder, and extract with 95% ethanol. Collected the filtrate and concentrate it on a rotary evaporator to acquire the crude ethanol extracts (KGEE). Dispersing KGEE in aqueous solution and petroleum ether, ethyl acetate, n-butanol solutions were used to fractionate KGEE successively. The fractions were collected separately and they were concentrated using a rotary evaporator to obtain the petroleum ether fraction (KGPE), ethyl acetate fraction (KGEA), n-butanol fraction (KGNB), and the remaining aqueous solution fraction (KGAS). Each fraction was subjected to screen activity by cell viability assay, and KGEA showed the best activity. Subsequently, the chemical identification of KGEA was conducted by ultra-high-performance liquid chromatography-quadrupole time-of-flight mass spectrometry (UHPLC-QTOF-MS) method.

### 2.3. LC-MS Analysis Conditions

UHPLC-QTOF-MS system (AB SCIEX) was applied to analyze KGEA. The analytical column used Waters ACQUITY UPLC BEH C18 (1.7 μm, 100 mm × 2.1 mm). A and B were mobile phases of 0.1% aqueous formic acid and acetonitrile. The flow rate was 0.2 mL/min and the gradient process was as follows: positive ion conditions: 0–4 min, 5~22% B; 4–12 min, 22~38% B; 12–20 min, 38~50% B; 20–30 min, 50~75% B; 30–35 min, 75~90% B; negative ion conditions: 0–3 min, 5~25% B; 3–13 min, 25~40% B; 13–18 min, 40~50% B; 18–35 min, 50~72% B; 35–46 min, 72~90% B. The injection volume was 5 μL. Mass spectrum conditions: electrospray ionization (ESI), ion source temperature 500 °C, scanning range 50–800, air curtain flow rate: 35 L/min, atomization gas flow rate: 50 L/min, auxiliary gas flow rate: 50 L/min, declustering potential: 100 V. The compounds were identified tentatively by examining their retention times and molecular weight and then comparing the MS spectral data generated by peakview (version 1.2) software.

### 2.4. Target Collection and Potential Target Prediction in KGEA

We obtained the SMILES (simplified molecular input line entry system) identifiers for the compounds identified by LC-MS by searching the Pubchem database (https://pubchem.ncbi.nlm.nih.gov (accessed on 22 June 2024)). SMILES numbers were imported into SwissADME (http://www.swissadme.ch/index.php (accessed on 22 June 2024)) for ADME (absorption, distribution, metabolism, excretion) parameter prediction. The compounds with high gastrointestinal absorption parameters and Lipinski rule of “yes” for >2 was examined. The Swiss Target Prediction tool (http://www.swisstargetprediction.ch/ (accessed on 23 July 2024)) was used to identify the targets of the selected compounds. GeneCards (https://www.genecards.org/ (accessed on 23 July 2024)) and OMIM (http://www.omim.org/ (accessed on 23 July 2024)) were utilized to identify genes associated with the keyword “oxidative”. A Venn diagram (http://bioinfogp.cnb.csic.es/tools/venny/ (accessed on 23 July 2024)) was used to determine which targets were overlapped between compounds and diseases.

### 2.5. Protein–Protein Interaction (PPI) of the Targets

Protein–protein interactions (PPI) among targets were determined via STRING (https://string-db.org/ (accessed on 23 July 2024)), with the biological species limited to “Homo sapiens” and an interaction score of 0.9. Utilizing CytoNCA (V2.1.6) plugin tool to obtain core targets and visualizing them using Cytoscape (version 3.8.2).

### 2.6. Functional Annotation and Pathway Analysis

DAVID (https://david.abcc.ncifcrf.gov/ (accessed on 24 July 2024)) was used to analyze GO (gene ontology) and KEGG (Kyoto encyclopedia of genes and genomes) enrichment and “Homo sapiens” was set as the selected species. The Weishengxin online tool (http://www.bioinformatics.com.cn/ (accessed on 24 July 2024)) facilitated the visualization of the top 10 GO functions, such as biological processes, cellular components, and molecular functions, along with KEGG pathways.

### 2.7. Component–Target–Pathway Network Construction

The network of component–target–pathway was composed of an active compound, target genes, and the pathway in which these genes participate. To construct this network for KGEA antioxidation, active ingredients, key targets, and top 20 KEGG pathways were input into Cytoscape 3.8.2 to analyze.

### 2.8. Molecular Docking

PubChem (https://pubchem.ncbi.nlm.nih.gov/ (accessed on 1 December 2024)) was used to obtain the 2D structure of the active ingredients and further processed to obtain the MOL2 format of them. The structure of the target protein was sourced from the PDB database (https://www.rcsb.org/ (accessed on 1 December 2024)). Autodock 1.5.7 was employed to analyze the binding interaction between compounds and proteins. The results of the docking were visualized through PyMOL (version 2.3) and MOE (version 2022) software.

### 2.9. Molecular Dynamics Simulation

MD simulations were conducted using GROMACS (version 5.1.5). The ligand topology file was generated using the AMBER force field via the ACPYPE script, while the protein topology file was created with the AMBER99SB-ILDN force field. A triclinic lattice incorporating TIP3P water molecules was employed for the simulation. Prior to the MD simulation, the system was neutralized with NaCl counterions and equilibrated through NVT and NPT ensembles for 100 ps. Each system was simulated under periodic boundary conditions at 300 K and 1.0 bar for 100 ns. Additionally, the binding free energy between proteins and ligands was calculated using the MM/GBSA method.

### 2.10. DPPH Radical Scavenging Assay

The DPPH assay was performed following the described method [23]. In brief, 50 μL of KGEA at varying concentrations (0.625, 1.25, 2.5, 5, and 10 mg/mL) was combined with 50 µL of 0.5 mM DPPH solution and incubated in the dark at room temperature for 30 min. As a positive control group, the concentrations of ascorbic acid were 5, 10, 20, 30, and 40 ug/mL. The absorbance was recorded at 517 nm using a microplate reader (TECAN, Switzerland). The free radical scavenging capacity was calculated using Equation (1):

Scavenging rate (%) = (*A*_0_ − *A*_1_)/*A*_0_ × 100% (1)

An absorbance value of *A*_0_ represents the absorbance of the control group without samples, while an absorbance value of *A*_1_ represents the absorbance of the KGEA group. At the same conditions, the scavenging rate of ascorbic acid were calculated.

### 2.11. ABTS Radical Scavenging Assay

The ABTS assay was performed according to reference [23]. Briefly, ABTS (7.4 mM) solution and K_2_S_2_O_8_ (2.45 mM) solution were mixed and left in the dark for 16 h to prepare the radical stock solution. Before use, the absorbance of the phosphate buffered saline (PBS)-diluted mixed solution to 734 nm was 0.70 ± 0.02. 50 μL of different concentrations of KGEA (0.4, 0.6, 0.8, 1.0, 1.2, and 1.4 mg/mL) were mixed with 150 µL of ABTS solution. The absorbance was measured at 734 nm using a microplate reader following a 6 min incubation at room temperature. The scavenging rate was calculated using Equation (1).

### 2.12. Cell Culture

RAW264.7 cells, sourced from iCell Bioscience Inc. (Shanghai, China), were cultured in DMEM medium containing 10% FBS and 1% penicillin-streptomycin solution. The cells were maintained in a 5% CO_2_ incubator at 37 °C.

### 2.13. Cell Viability Assay

Cell viability was assessed using the CCK-8 assay. RAW264.7 cells were seeded in 96-well plates at a density of 1 × 10^4^ cells/well and incubated overnight. The cells were then treated with varying concentrations of KGEE, KEPE, KGEA, KGNB, and KGAS (2, 4, and 8 μg/mL) for 24 h. After adding 10 µL of CCK-8 solution to each well, absorbance was measured at 450 nm using a microplate reader.

Investigating the protective effects of different fractions on H_2_O_2_-induced cell damage, cells were treated with KGEE, KEPE, KGEA, KGNB, and KGAS (2, 4 and 8 μg/mL) for 20 h, followed by addition of H_2_O_2_ for 4 h. CCK-8 solution was then added for detection.

### 2.14. ROS Assay

ROS assay kit was used to detect ROS generation following the manufacturer’s protocol. Cells were placed in 6-well plates with different KGEA concentrations, and after treatment, DCFH-DA diluted at a ratio of 1:1000 was added to each well and incubated for 30 min. The examination of fluorescence intensity was conducted with a fluorescence microscope.

### 2.15. JC-1 Assay

The mitochondrial membrane potential was assessed using a commercial JC-1 kit, with cells exposed to a range of KGEA concentrations. The cells were incubated for 24 h, washed once with PBS, and then treated with JC-1 working solution for 20 min at 37 °C in the dark. The JC-1 dye was taken out, and the cells in the medium were examined with a fluorescence microscope.

### 2.16. Evaluation of Antioxidant Enzyme Activity and Lipid Peroxidation

RAW264.7 cells were spread into 6-well plates at a density of 1 × 10^6^/well and incubated for 24 h. The cells were treated with KGEA at different concentrations for 20 h and induced with H_2_O_2_ (400 µM) for 4 h. An evaluation of the activities of CAT, GSH-Px, MDA, and SOD in cells was conducted using a commercial kit.

### 2.17. Western Blotting Analysis

The supernatants from the cells were taken out, and the cells were rinsed with PBS. RIPA buffer was used to lyse the cells and extract total protein. Subsequently, protein content was assessed using BCA kits (Beyotime Biotechnology Co., Ltd., Shanghai, China). The proteins from the cell lysates were separated using SDS-PAGE (Shanghai Yamay Biomedical Technology Co., Ltd., Shanghai, China) and the proteins were then transferred onto nitrocellulose (NC) membranes. Following incubation with primary and secondary antibodies, the NC membrane was examined after being treated with an ECL (enhanced chemiluminescence) solution. The optical density of the bands was quantified using ImageJ (version 1.51) software.

### 2.18. Zebrafish Husbandry

For this experiment, a wild-type AB zebrafish was obtained from the Guangdong Laboratory Animals Monitoring Institute and maintained at 28.5 ± 0.5 °C with a cycle of 14:10 h light/dark in an incubator. Collected embryos from natural spawning, which was induced by light. The embryos were cultured in E3 medium. The zebrafish experiments have approved and conducted by the Guangdong Provincial Biotechnology Research Institute (No. IACUC2022107).

### 2.19. Waterborne Exposure of Zebrafish Embryos to KGEA and H_2_O_2_

The protective effect of KGEA on H_2_O_2_-induced zebrafish embryotoxicity was evaluated. Different concentrations of KGEA (2, 4, and 8 µg/mL) treated the 48 h post-fertilization (hpf) embryos for 1 h. Incubation was continued for 24 h with H_2_O_2_ (2 mM). The survival rate was then determined.

### 2.20. Heartbeat Rate of Zebrafish Embryos

Zebrafish embryos were exposed to KGEA (2, 4, and 8 µg/mL) for 1 h, before H_2_O_2_ (2 mM) was administered. Place zebrafish embryos under a microscope for observation after 24 h of incubation (Nikon Ci-E, Minato-ku, Japan). Heartbeat rate of zebrafish embryos was determined by manually counting every 10 s.

### 2.21. Determination of KGEA Against H_2_O_2_-Induced Oxidative Stress in Zebrafish Embryos

The ROS levels in zebrafish embryos were quantified using DCF-DA. A 2.5 µM DCF-DA solution was added to incubate with the embryos for 50 min. After washing with E3 medium and anesthetizing with MS222 (150 µg/mL), images were taken using a fluorescence microscope (Nikon Ci-E, Japan).

Zebrafish embryo cell death was assessed with acridine orange. Formation of pigment pattern in zebrafish was inhibited by phenylthiourea (2 mg/mL) for better fluorescence observation. Various concentrations of KGEA were administrated for 1 h, and H_2_O_2_ (2 mM) was added followed. Incubation for 24 h and E3 medium was used to wash the embryos. Acridine orange (7 µg/mL) was added, and the embryos were incubated in the dark for 0.5 h. The embryos were washed with E3 medium and MS222 was employed to anesthetize them. Embryo images were captured using a fluorescence microscope.

The lipid peroxidation of zebrafish embryos was determined by DPPP. A solution of DPPP at 20 µg/mL was given and incubated in the dark for a duration of 40 min. Then, the embryos were anesthetized, and images were photographed.

### 2.22. Determination of Antioxidant Enzyme Activity and Lipid Peroxidation in Zebrafish Embryos

Different concentrations of KGEA were administrated for 1 h, and H_2_O_2_ (2 mM) was added for incubation 24 h. Embryos from each group were collected and homogenized in PBS using a tissue homogenizer (1700 r/min, 30 s × 3). The supernatants were collected after discarding the precipitate. The activities of SOD, CAT, GSH-Px, and MDA levels in the embryos were measured using commercial assay kits.

### 2.23. Statistical Analysis

Data analysis was performed using SPSS 26.0 software with a one-way ANOVA test, followed by either the least significant difference (LSD) or Dunnett’s multiple comparisons test. Results are presented as means ± SD, with *p* < 0.05 considered statistically significant.

## 3. Results

### 3.1. Discovery of Bioactive Fraction in K. galanga

**Figure 1:**
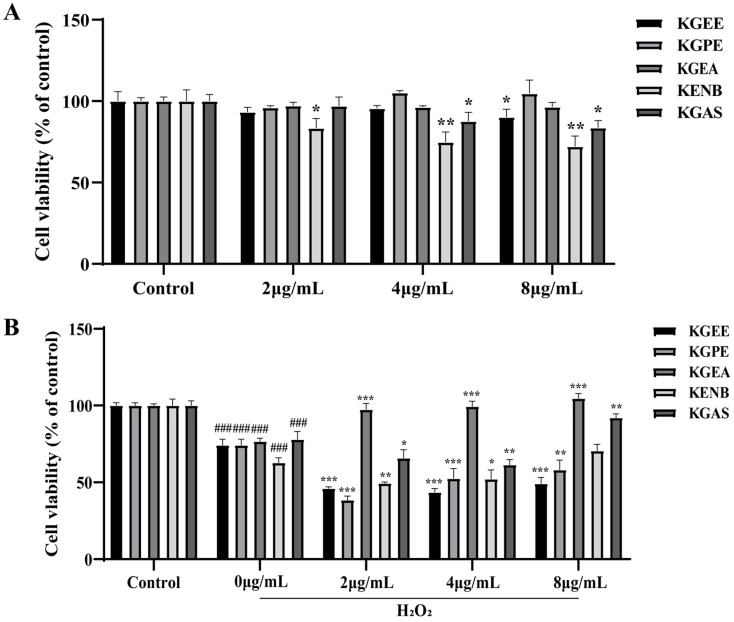
Effects of each fraction of ethanol extract from *K. galanga* on the viabilities of H_2_O_2_-treated and untreated RAW 264.7 cells. (**A**) Cell viability of H_2_O_2_-untreated cells. (**B**) Cell viability of H_2_O_2_-induced cells. Data are displayed as mean ± SD. ### *p* < 0.001 vs. control group, * *p* < 0.05, ** *p* < 0.01 and *** *p* < 0.001 vs. H_2_O_2_ group.

We first obtained the 95% ethanol extracts of *K. galanga* (KGEE). Then, the KGEE was further extracted by different polar solvents to obtain the petroleum ether fraction (KGPE), ethyl acetate fraction (KGEA), n-butanol fraction (KGNB), and the remaining aqueous solution fraction (KGAS). The effects of different concentrations of KGEE and each fraction of KGEE on cell viability were measured using the CCK-8 assay. As shown in Figure 1A, KGEE, KGPE, and KGEA showed no significant inhibition of cells viability at concentration of 2, 4, and 8 ug/mL, and KGNB, KGAS showed inhibitory effects. Moreover, the protective effects of them on H_2_O_2_-treated cells were evaluated. In Figure 1B, cell viability in untreated cells was considered as 100%, and in the H_2_O_2_-traeted group, cell viability was reduced obviously. However, KGEA could enhance cell viability of H_2_O_2_-traeted cells at a concentration of 2 ug/mL; furthermore, with the increase in drug concentration, the cell viability of H_2_O_2_-induced cells was increased. This denoted KGEA had a better protective effect on H_2_O_2_-induced cell damage. Therefore, KGEA was chosen for following experiments.

### 3.2. LC-MS Analysis of the KGEA

The chemical composition of KGEA was analyzed by UPLC-Q-TOF-MS/MS in positive and negative ion mode conditions (Supplementary Figure S1). The compounds were identified by analyzing the MS spectra with PeakView software (version 2.0) and validating the results through comparison with the literature data [24,25,26,27,28]. A total of 43 known components were determined, which included terpenoids, phenols, flavonoids, and others (Table 1). [M − H]^−^.

### 3.3. Potential Targets of KGEA Compounds and Antioxidants

**Figure 2:**
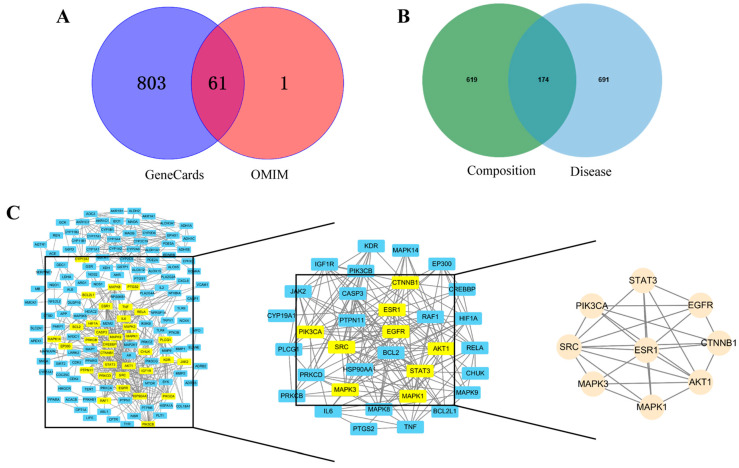
Target determination and protein–protein interaction (PPI) network analysis. (**A**) Venn diagram of oxidative targets. (**B**) Venn diagram of potential target for the action of KGEA and oxidative. (**C**) Protein–protein interaction (PPI) network of KGEA as a target for antioxidant treatment.

From the 43 compounds in KGEA, 25 were identified through the SwissADME database, satisfying the conditions of “high” gastrointestinal absorption and drug-likeness marked as “Yes” for over two items. A total of 793 potential targets for 25 compounds were predicted using Swiss Target Prediction. The number of oxidative targets selected from OMIM and GeneCards were 62 and 864 (Figure 2A), using “oxidative” as the keyword. Through Venn analysis, 174 common targets were obtained from compounds and disease targets (Figure 2B).

### 3.4. PPI Network of the Targets

Key targets were revealed by using the STRING database with the criteria of score 0.9. As displayed in Figure 2C, the PPI network of the common targets was visualized with Cytoscape software (version 3.9.1), and the key nine genes (SRC, STAT3, AKT1, ESR1, MAPK1, MAPK3, PIK3CA, CTNNB1, EGFR) were screened out.

### 3.5. GO Enrichment and KEGG Pathway Analyses

**Figure 3:**
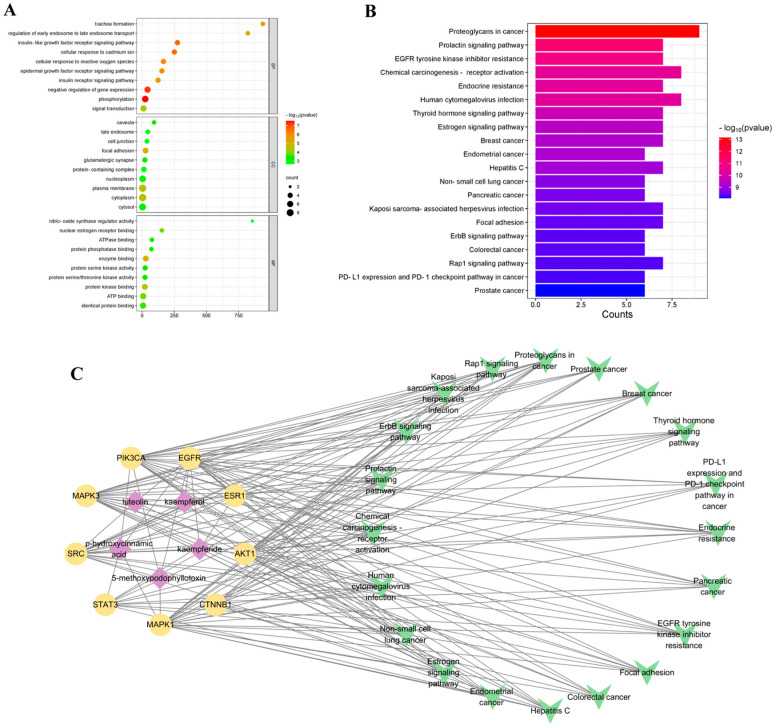
GO and KEGG enrichment analysis of target genes, and establishment of the component–target–pathway network. (**A**) GO enrichment analysis of the top 10. (**B**) KEGG pathway enrichment analysis of the top 20. (**C**) The component–target–pathway network of KGEA.

The nine key targets were analyzed for GO and KEGG enrichment using the DAVID database. GO analysis showed that a total of 147 GO terms were selected with the *p*-value parameter (*p* < 0.05), which contained molecular functions (MF), biological processes (BP), and cellular components (CC). As shown in Figure 3A, the terms ranked top 10 of BP, CC, and MF were arranged. The biological processes mainly involved in trachea formation, regulation of early endosome to late endosome transport and insulin-like growth factor receptor signaling pathway. The cellular components were mainly associated with caveola, late endosome and cell junction. The molecular functions primarily participate in nitric- oxide synthase regulator activity, nuclear estrogen receptor binding, and ATPase binding.

Moreover, KEGG enrichment analysis was carried out (Figure 3B), and 108 pathways (*p* < 0.05) were obtained. The pathways ranked top 20 of KEGG were screened out, and that mainly related to proteoglycans in cancer, prolactin signaling pathway and EGFR tyrosine kinase inhibitor resistance.

### 3.6. Component–Target–Pathway Network Analysis

The active compounds, key targets and signaling pathways were analyzed. As revealed in Figure 3C, luteolin, kaempferide, kaempferol, 5-methoxypodophyllotoxin and p-hydroxycinnamic acid were the key ingredients that may contribute to the antioxidant effect of KGEA. SRC, STAT3 and AKT1 were the top three key targets. The pathway map of KGEA was obtained by using KEGG mapper tool. The component–target–pathway network was assembled using Cytoscape. The results show that the antioxidant targets of KGEA were related to multiple pathways among proteoglycans in cancer, prolactin signaling pathway, and EGFR tyrosine kinase inhibitor resistance, and that KGEA mainly regulated these pathways to exert function. Targets include ESR1, MAPK1, and AKT1.

### 3.7. Molecular Docking Analysis

**Figure 4:**
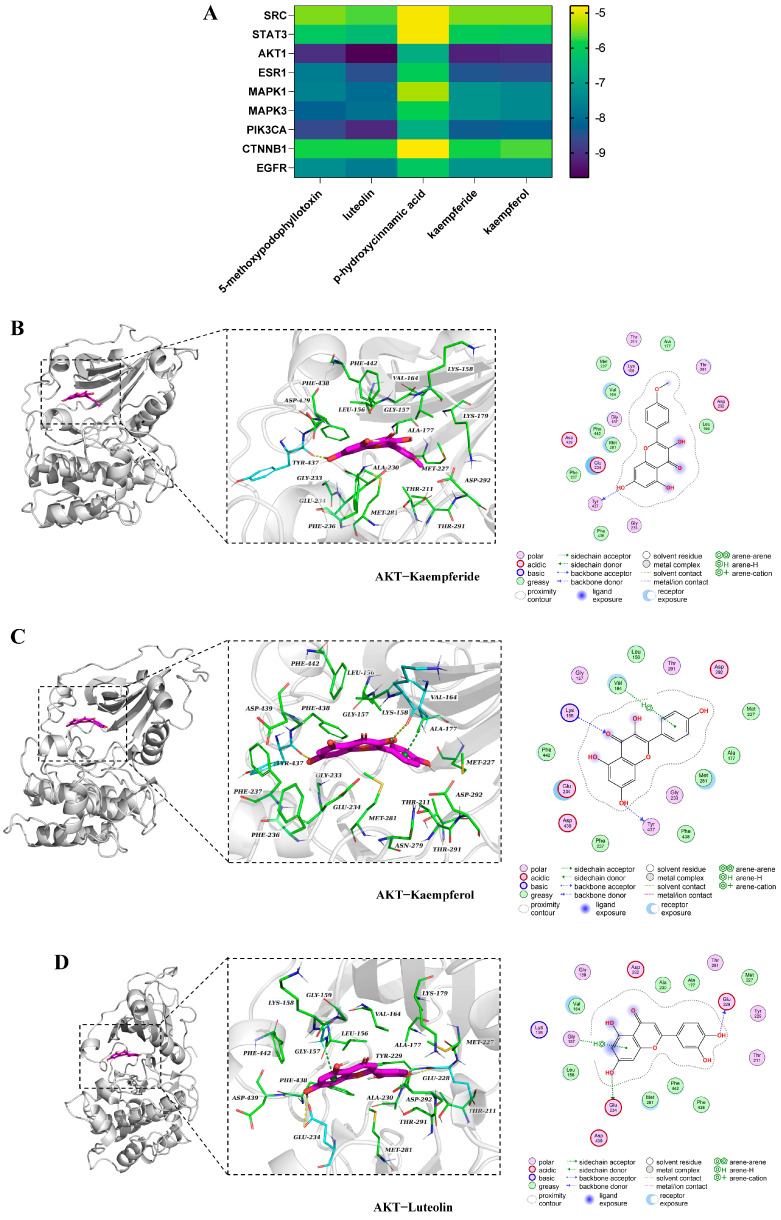
Molecular docking analysis of AKT1−Kaempferide, AKT1−Kaempferol and AKT1−Luteolin complexes. (**A**) Heat map analysis of active ingredients and key targets. Binding modes of AKT1 and ligands. (**B**) A 3D close view and 2D interactions of ATK1 and Kaempferide. (**C**) A 3D close view and 2D interactions of ATK1 and Kaempferol. (**D**) A 3D close view and 2D interactions of ATK1 and Luteolin.

Molecular docking was conducted between the five active ingredients and nine key targets, and the degree of the docking results are shown in Figure 4A. Scores below −5.0 kJ/mol indicate high binding affinity, while scores below −7.0 kJ/mol suggest a strong interaction between the compound and target protein [29]. The binding affinity between kaempferide and AKT1 was −9.2 kJ/mol, forming hydrogen bonds with TYR437 (Figure 4B). Kaempferol had a stable binding affinity of −9.1 kJ/mol and displayed hydrogen bonding with TYR437, LYS158, and Pi-Pi stacking with VAL164 (Figure 4C). As shown in Figure 4D, luteolin was strongly bound with AKT1 with a binding affinity of −9.7 kJ/mol, and formed hydrogen bonds with GLU228, GLU234, and Pi-Pi stacking with GLY157.

### 3.8. Molecular Dynamics Simulation Analysis

**Figure 5:**
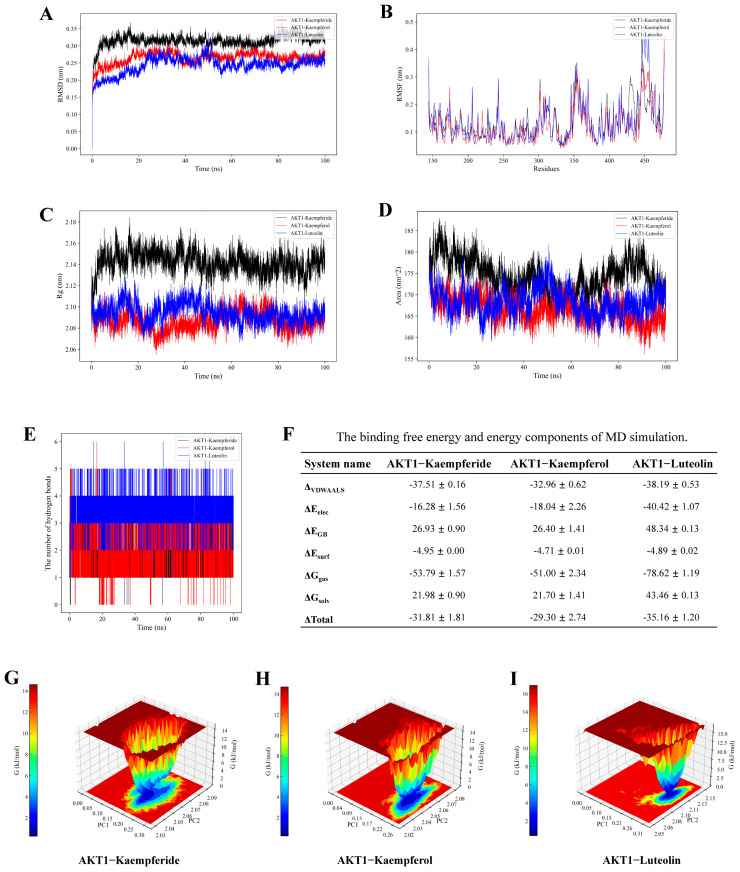
MD simulation analysis of AKT1−Kaempferide, AKT1−Kaempferol, and AKT1−Luteolin complexes. (**A**) RMSD. (**B**) RMSF. (**C**) Rg. (**D**) The number of hydrogen bonds. (**E**) SASA. (**F**) The binding free energy (kcal/mol) and energy components of MD simulation. (**G**) The Gibbs energy landscape of AKT1−Kaempferide. (**H**) The Gibbs energy landscape of AKT1−Kaempferol. (**I**) The Gibbs energy landscape of AKT1−Luteolin.

The root-mean-square deviation (RMSD) was utilized to evaluate the stability of the receptor–ligand complex. The RMSD curves for the AKT1−Kaempferide, AKT1−Kaempferol, and AKT1−Luteolin complexes were stable within the ranges of 0.28–0.35 ns, 0.22–0.28 ns, and 0.20–0.25 ns, respectively, with the AKT1−Luteolin complex demonstrating greater stability (Figure 5A).

Root-mean-square fluctuation (RMSF) serves as an indicator of the complex’s fluctuation at the residue level. The flexibility of residues 150–300 was higher in the AKT1−Kaempferide complex than in the AKT1−Kaempferol complex, and residues 150–400 in the AKT1−Luteolin complex were of lower residue flexibility (Figure 5B).

The radius of gyration (Rg) represents the system’s binding tightness and constraints, indicating the degree of protein folding [30]. Figure 5C demonstrates that the Rg values for the AKT1−Kaempferide, AKT1−Kaempferol, and AKT1−Luteolin complexes were stable in the ranges of 2.14–2.18 nm, 2.08–2.10 nm, and 2.08–2.11 nm.

The solvent accessible surface area (SASA) was computed to estimate the interactions between the complex and the solvents in the media. As illustrated in Figure 5D, the AKT1−Kaempferol complex showed the lowest average SASA value and exhibited greater stability.

As a strong non-covalent interaction, hydrogen bonding was observed in the AKT1−Kaempferide complex with 0–2 bonds in 0–100 ns (Figure 5E), and in the AKT1−Kaempferol complex with 1–3 bonds. The AKT1−Luteolin complex formed a maximum of 3–5 hydrogen bonds.

The binding free energies and energy components of the three complexes are shown in Figure 5F.

The Gibbs energy landscape illustrates the intricate stability. As depicted in Figure 5G–I, The AKT1−Luteolin complex had more stable free energy when the Rg value ranged from 2.08 to 2.12 and the RMSD value was between 0.25 and 0.30.

### 3.9. DPPH Radical Scavenging Activity

The DPPH radical scavenging assay was employed to assess the overall free radical scavenging capacity of substances. DPPH, a stable free radical, exhibits reduced absorbance when it accepts an electron or hydrogen radical from antioxidants, allowing its scavenging ability to be measured. The DPPH free radical scavenging activity of KGEA was evaluated. The results show that KGEA had potent DPPH radical scavenging effect, and its EC50 value was 4.88 ± 0.74 mg/mL (Table 2).

### 3.10. ABTS Radical Scavenging Activity

When a single electron and combing with hydrogen (H^+^) was accepted by ABTS radical (ABTS^+^), the stable ABTS is formed. This process plays a significant role in free radical scavenging. To determine the antioxidant capacity of KGEA, the ABTS radical scavenging assay was also applied. In Table 2, the scavenging activity of KGEA with various concentrations was detected, and its IC_50_ was 1.07 ± 0.06 mg/mL.

### 3.11. KGEA Alleviated Oxidative Stress in H_2_O_2_-Induced RAW264.7 Cells

**Figure 6:**
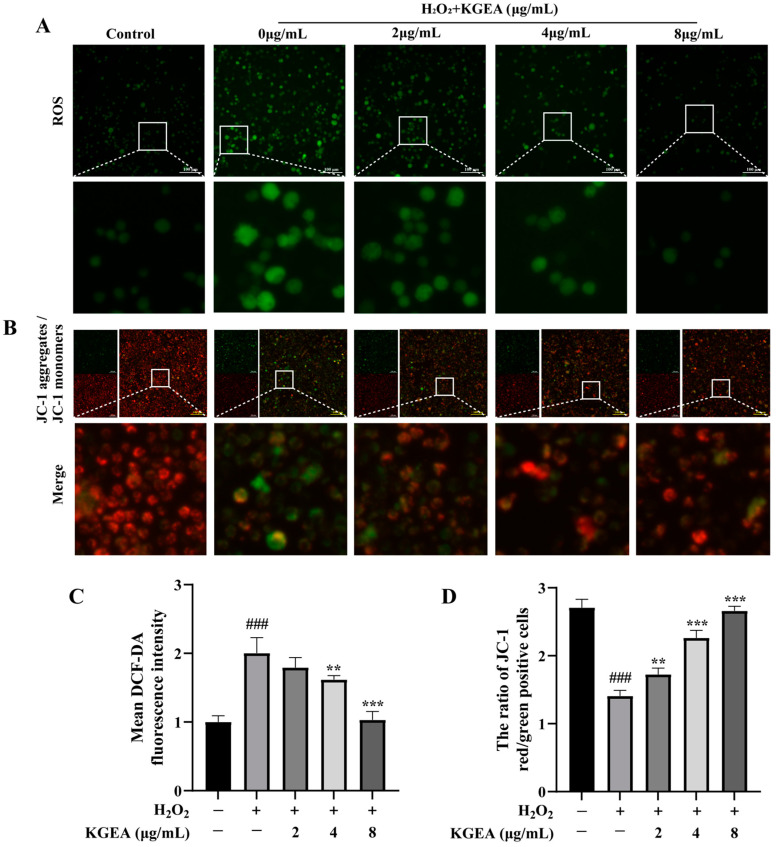
ROS and mitochondrial membrane potential analysis in H_2_O_2_-induced RAW264.7 cells. (**A**) ROS level and (**B**) mitochondrial membrane potential level were visualized by fluorescence microscopy. (**C**) The ROS data. (**D**) The mitochondrial membrane potential data. Data are displayed as mean ± SD. ### *p* < 0.001 vs. control group, ** *p* < 0.01 and *** *p* < 0.001 vs. H_2_O_2_ group.

The relative fluorescence intensity of DCF-DA in each group was measured to assess ROS concentration (Figure 6A). Compared to the control groups, the cell fluorescence intensity in the H_2_O_2_ groups was significantly increased. The KGEA-treated groups showed significantly lower intracellular fluorescence intensities than the H_2_O_2_ group (*p* < 0.05), indicating that KGEA can reduce intracellular ROS levels.

The mitochondrial membrane potential is crucial for sustaining proper mitochondrial function and cellular activity. A reduction in mitochondrial membrane potential signals the onset of early apoptosis. Our findings indicate that the red/green fluorescence ratio in the H_2_O_2_ groups was notably lower than in the control groups, suggesting that the H_2_O_2_ groups decreased membrane potentials. In contrast to the H_2_O_2_ groups, the KGEA treatment notably raised the red/green fluorescence ratio, indicating that KGEA reduced mitochondrial damage (Figure 6B).

### 3.12. KGEA Promoted Antioxidant Enzyme Activity and Reduced Lipid Peroxidation

**Figure 7:**
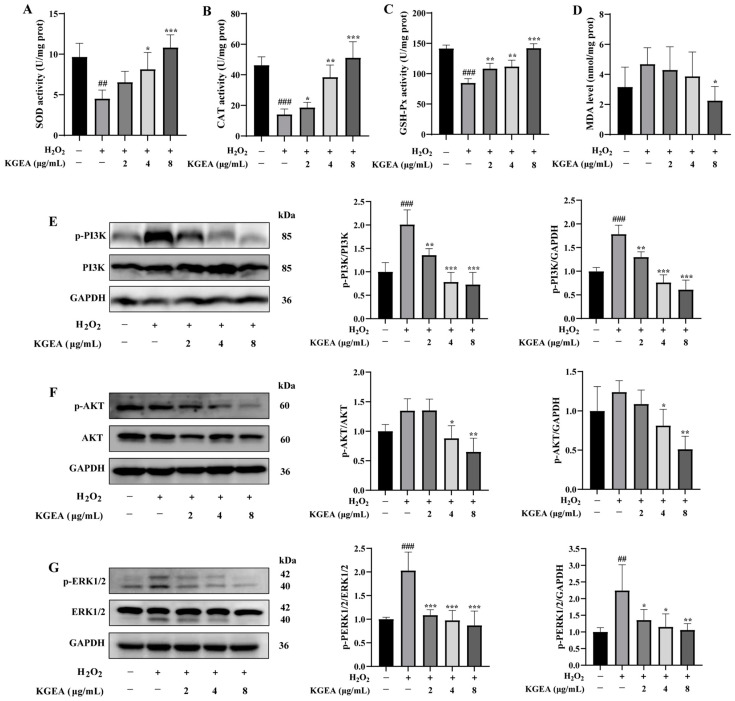
Effects of KGEA on intracellular antioxidant defense system and PI3K/AKT, MAPK pathways in H_2_O_2_-induced RAW 264.7 cells. (**A**–**D**) The activity of SOD, CAT, GSH-Px, MDA, respectively. (**E**–**G**) Protein expression in each group. Data are displayed as mean ± SD. ## *p* < 0.01, ### *p* < 0.001 vs. control group, * *p* < 0.05, ** *p* < 0.01 and *** *p* < 0.001 vs. H_2_O_2_ group.

To further explore KGEA’s role in mitigating oxidative stress, its effects on antioxidant enzyme activity and lipid peroxidation in H_2_O_2_-induced cells were assessed. As shown in Figure 7A–C, the activities of SOD, CAT, and GSH-Px were significantly lower in the H_2_O_2_-treated group compared to the control group. However, KGEA pretreatment notably enhanced these antioxidant enzyme activities. In Figure 7D, MDA levels rose significantly after H_2_O_2_ induction but decreased markedly in the KGEA-treated groups. These results indicate that KGEA exerted antioxidant effects by boosting GSH-Px, CAT, and SOD activities while reducing MDA levels.

### 3.13. Inhibition of KGEA on H_2_O_2_-Induced PI3K/AKT and MAPK Pathways

To investigate the activation of oxidative-stress-related pathways induced by H_2_O_2_, measurements of p-PI3K, p-AKT, and p-ERK1/2 levels were taken (Figure 7E–G). The data revealed that the expression of p-PI3K and p-AKT proteins was slightly increased in the H_2_O_2_ groups and markedly decreased in the KGEA groups, as revealed in Figure 7E–G. In addition, KGEA inhibited the ERK1/2 phosphorylation compared with the H_2_O_2_ group. This indicates that the protection of cells from oxidative damage by KGEA is achieved through the regulation of the PI3K-AKT and MAPK signaling pathway.

### 3.14. Protective Effects of KGEA in an H_2_O_2_-Induced Zebrafish Model

**Figure 8:**
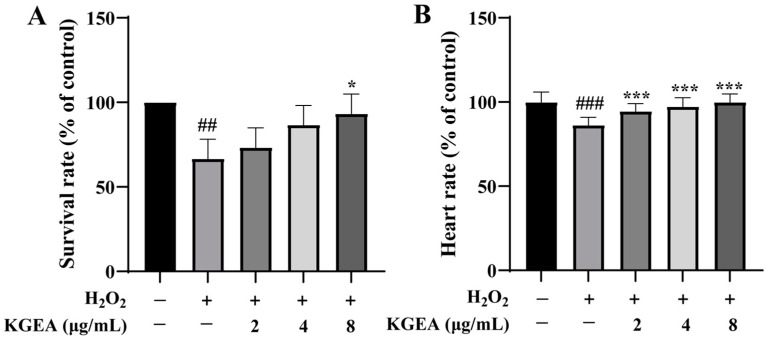
Survival rate and heartbeat rate of H_2_O_2_-induced zebrafish treated with KGEA. The survival rate (**A**) and the heartbeat (**B**) of zebrafish. The results are expressed as mean ± SD. ## *p* < 0.01, ### *p* < 0.001 vs. control group. * *p* < 0.05, *** *p* < 0.001 vs. H_2_O_2_ group.

The antioxidant activity of KGEA in vivo was evaluated using an H_2_O_2_-induced zebrafish model. As displayed in Figure 8A, the survival rate of zebrafish significantly decreased when exposed to H_2_O_2_. Meanwhile, KGEA administration could obviously enhance this survival rate. In addition, the heartbeat rate of zebrafish decreased with exposure to H_2_O_2_, while with pretreatment with KGEA, the heartbeat rate improved (Figure 8B).

### 3.15. Antioxidant Effects of the KGEA in an H_2_O_2_-Induced Zebrafish Model

**Figure 9:**
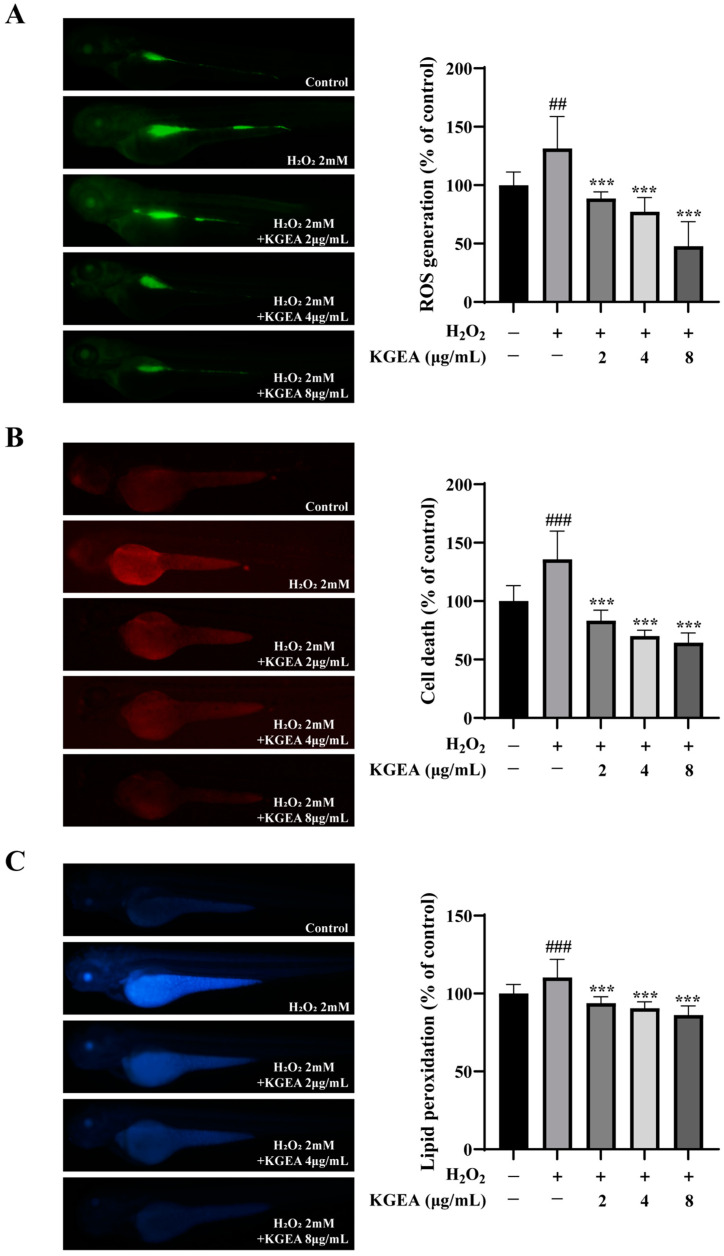
The effects of KGEA on H_2_O_2_-induced ROS Production, cell death, and lipid peroxidation in zebrafish. Zebrafish were co-cultured with or without KGEA and stimulated with or without H_2_O_2_; ROS generation (**A**), Cell death (**B**), and lipid peroxidation (**C**) were determined. The data are expressed as the mean ± SD. ## *p* < 0.01, ### *p* < 0.001 vs. control group. *** *p* < 0.001 vs. H_2_O_2_ group.

To further observe the antioxidant effects of KGEA, ROS levels, cell death, and lipid peroxidation were evaluated by fluorescence probe methods in the zebrafish model. As presented in Figure 9A, the ROS levels were increased in the H_2_O_2_-induced group, which was decreased in those co-cultured with KGEA groups. In Figure 9B, zebrafish were exposed to H_2_O_2_, and the cell death increased obviously. When given different concentrations of KGEA, cell death reduced. In Figure 9C, lipid peroxidation in zebrafish was induced by H_2_O_2_, while it was reduced after KGEA treatment. The results indicate that KGEA could protect zebrafish from oxidative stress damage induced by H_2_O_2_, which was associated with the reduction in ROS levels, lipid peroxidation, and relieving cell death.

### 3.16. Antioxidant Effects of KGEA on Antioxidant Enzymes Activities and MDA Level in an H_2_O_2_-Induced Zebrafish Model

**Figure 10:**
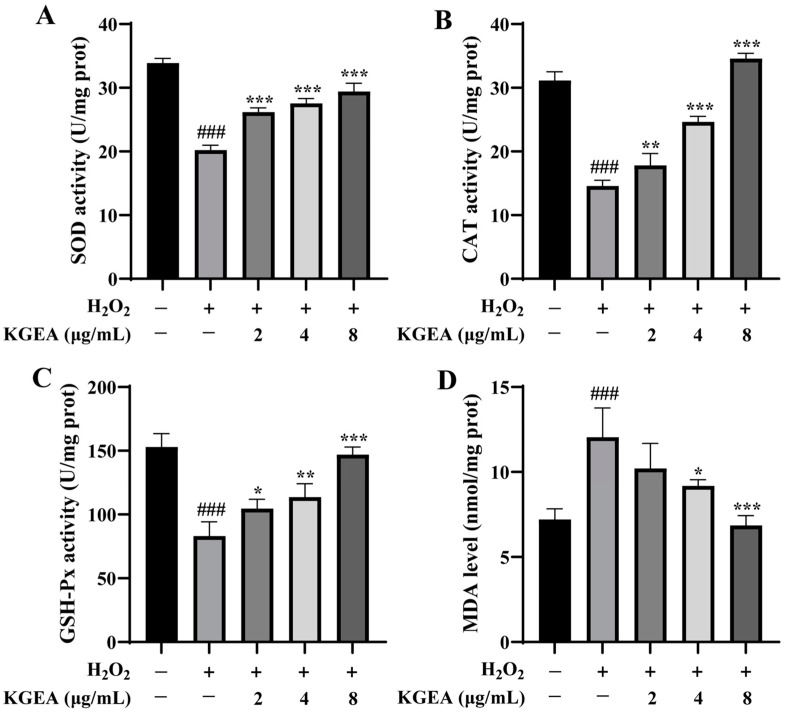
The effects of KGEA on antioxidant enzymes activities and MDA levels in H_2_O_2_-induced zebrafish. The activities of SOD (**A**), CAT (**B**), GSH-Px (**C**), and MDA levels (**D**) were determined. The data are expressed as mean ± SD. ### *p* < 0.001 vs. control group. * *p* < 0.05, ** *p* < 0.01, *** *p* < 0.001 vs. H_2_O_2_ group.

The antioxidant effects of KGEA (2, 4, and 8 µg/mL) were evaluated using the H_2_O_2_-induced zebrafish model, and the antioxidant enzyme activities were evaluated. As displayed in Figure 10A–C, in zebrafish embryos, the H_2_O_2_-induced group had lower SOD, CAT, and GSH-Px activities than the control group. Meanwhile, in the pretreatment group of KGEA, the antioxidant enzyme activities increased with the increase in drug concentration. The MDA levels significantly increased after H_2_O_2_ stimulation, and in the KGEA administration groups, the MDA levels decreased (Figure 10D). The results indicate that H_2_O_2_-induced oxidative stress, which were related to decreasing antioxidant enzyme activities and increasing MDA levels, while treatment of KGEA could alleviate damage.

## 4. Discussion

Oxidative stress contributes to the development and progression of various diseases by damaging biomolecular and cellular structures and affecting the normal functions of organs and systems [31]. In light of its key role in a range of diseases, the development of effective antioxidant treatments for oxidative stress is important. Modern research has reported that traditional Chinese medicines (TCM) contain diverse natural antioxidants [32]. *K. galanga* is a well-known TCM with antioxidant, antimicrobial, anti-inflammatory, and antitumor activities [12,13,14,15]. However, its antioxidant activity and related mechanisms have received extremely limited attention. In this study, we used 95% ethanol to extract *K. galanga*, and the ethanol extract (KGEE) was obtained. Then, the KGEE was subjected to further fractionation using various polar solvents, yielding the petroleum ether fraction (KGPE), ethyl acetate fraction (KGEA), n-butanol fraction (KGNB), and the remaining aqueous solution fraction (KGAS). We screened effective antioxidant fraction KGEA, studied its chemical composition, antioxidant activity, and potential antioxidant mechanisms.

In cell viability assays, KGEA did not influence cellular activity. Additionally, a standard oxidative stress model was utilized, wherein RAW264.7 cells were exposed to H_2_O_2_, and this model was implemented effectively. KGEA demonstrated a significant protective effect against H_2_O_2_-induced oxidative stress damage in cells, in comparison to KGEE, KGPE, KGNB, and KGAS. Thus, KGEA was chosen as the most antioxidant fraction in *K. galanga* for further studies.

The chemical composition of KGEA was analyzed using LC-MS technology, resulting in the identification of 43 chemical components. Following screening via the Swiss ADME database, 25 compounds from KGEA were selected for network pharmacological analysis, leading to the identification of 793 compound targets and 865 antioxidant targets. Venn analysis revealed 174 common targets between the compound and antioxidant targets. 5-methoxypodophyllotoxin, luteolin, p-hydroxycinnamic acid, kaempferide, and kaempferol were identified as key antioxidant components in KGEA by component–target–pathway network analysis. The antioxidant effect of key compounds luteolin, p-hydroxycinnamic acid, and Kaempferol have been reported [33,34,35,36]. Moreover, bisdemethoxycurcumin, linoleic acid, and vanillic acid exhibited potential antioxidant activity [37,38,39]. Further research is required to investigate the other chemical components that have not yet been reported.

Furthermore, key targets such as STAT3, PIK3CA, MAPK3, ESR1, SRC, and AKT1 were primarily involved in the PI3K-AKT and MAPK signaling pathways. Combining analysis of GO and KEGG enrichment found that these genes were mainly related to proteoglycans in cancer, the prolactin signaling pathway, EGFR tyrosine kinase inhibitor resistance, and the Rap1 signaling pathway. This suggests that oxidative stress was implicated in multiple diseases, and antioxidant mechanisms represented a promising strategy for preventing cancer and related conditions.

For the component–target–pathway network, luteolin, kaempferide, kaempferol, 5-methoxypodophyllotoxin and p-hydroxycinnamic acid were the key ingredients. We analyzed Figure S1 (Supplementary Material) and speculated that the content of these five chemical components was relatively low according to their peak areas. We presumed that these five components were the components with strong antioxidant effect in KGEA, but due to the low content of them, the contributions of them to the antioxidant activity of KGEA may not be the most significant. If the content of them in KGEA could be increased, the antioxidant activity of KGEA may be increased. In the follow-up study, we consider improving the extraction process to increase the content of these components in KGEA and significantly improve the antioxidant activity of KGEA.

Molecular docking and MD simulation analysis were applied to illuminate the mechanism and provide guidance to screen potential drugs. Based on the results, it showed that luteolin, kaempferide, and kaempferol were good choices for interactions with AKT1. Luteolin exhibits antioxidant activity, eliminating free radicals and enhancing antioxidant enzyme activity [40]. It was found that kaempferide had antioxidants to promote osteogenesis effectively [41]. Furthermore, studies show that kaempferol has antioxidant properties [42].

To investigate the antioxidant activity of KGEA in vitro, DPPH and ABTS scavenging activities were detected. The reducing power of a substance could be evaluated through the ability to accept electrons provided by DPPH [43]. The ABTS assay was employed to detect the kinetics of enzymes related to the production of H_2_O_2_ indirectly [44]. Results show that KGEA has strong DPPH and ABTS scavenging activities.

Elevated levels of ROS can lead to tissue damage [45]. We observed that KGEA has a protective role against oxidative damage. Elevated ROS can result in mitochondrial and cellular dysfunction, metabolic disorders, and apoptosis [46]. Our study found that KGEA was effective in preserving mitochondrial membrane potential after ROS damage.

To mitigate oxidative stress, the primary mechanisms involve enzymatic antioxidant defenses, primarily regulated by SOD, CAT, and GSH-Px [47]. Lipid peroxides are formed through the interaction of oxygen free radicals with unsaturated fatty acids, subsequently decomposing into various compounds, including MDA. The concentration of MDA serves as an indicator of cellular lipid oxidation levels [48]. The administration of KGEA at a concentration of 2, 4, and 8 µg/mL enhanced the activities of the antioxidant enzymes SOD, CAT, and GSH-Px. Furthermore, KGEA demonstrated an inhibitory effect on MDA levels within cells.

In this study, H_2_O_2_-induced activation of AKT and PI3K was noted in cells, and KGEA treatment markedly suppressed their phosphorylation. Additionally, the phosphorylation level of ERK1/2 was lowered by KGEA treatment. This implies that KGEA could be involved in oxidative stress damage caused by H_2_O_2_ through the regulation of PI3K, AKT, and ERK1/2. KGEA may exert its antioxidant effects by dual-targeting PI3K/AKT and MAPK pathways downstream of Rap1 signaling pathway, thereby fortifying cellular antioxidant defenses.

The zebrafish model was employed to investigate the in vivo antioxidant activity of KGEA. Upon exposure to H_2_O_2_, a significant decrease in the survival rate of zebrafish was observed. However, treatment with KGEA nearly restored the survival rate to normal levels and improved heart rate. Moreover, KGEA alleviated oxidative stress damage by reducing ROS production, preventing cell death, and mitigating lipid peroxidation induced by H_2_O_2_.

During oxidative stress, the enzymes SOD, CAT, and GSH-Px are regarded as crucial components of the defense mechanism. Redundant ROS caused lipid peroxidation, and MDA is seen as an indicator of that [49]. KGEA could significantly enhance enzyme activity and decrease the MDA level, which was associated with alleviating oxidative stress damage caused by H_2_O_2_.

This study indicates that KGEA exerted antioxidant effects related to multiple ingredients, targets, and pathways. These findings suggest that KGEA holds potential for development as a natural antioxidant.

## 5. Conclusions

This study adopts multiple antioxidant activity tests in vitro and in vivo, which indicate that KGEA could eliminate free radicals and reduce oxidative stress damage. The protective action of KGEA was mainly related to its inhibition of the PI3K-AKT and MAPK signaling pathways. Nevertheless, investigating the precise molecular mechanisms and advancing studies for clinical applications is necessary. These findings indicate that KGEA has the potential to develop as a novel therapeutic agent for combating antioxidant diseases.

## Figures and Tables

**Table 1:** Compounds of KGEA.

No.	RT (min)	Molecular Name	Molecular Formula	[M + H]^+^	[M − H]^−^	Fragment Ions (*m*/*z*)
1	5.21	4-methoxybenzyl-O-β-D-glucopyranoside	C_14_H_20_O_7_	301.1282		205.0153, 160.9889
2	8.26	bisdemethoxycurcumin	C_19_H_16_O_4_	309.1134		217.0764
3	9.37	ponkanetin	C_20_H_20_O_7_	373.1295		131.0482, 147.0440, 163.0751, 175.0756
4	10.01	3-caren-5-one	C_10_H_14_O	151.1117		109.0640, 107.0494
5	12.70	feruloylputrescine	C_14_H_20_N_2_O_3_	265.1554		265.0985, 247.0877, 235,0876, 219.0926, 218.0849, 206.0843, 167.0672
6	16.14	dehydrocholic acid	C_24_H_34_O_5_	403.2493		117.0697, 373.0926
7	19.51	3′,4′,5,7-tetramethylquercetin	C_19_H_18_O_7_	359.1143		359.2045, 197.0978, 177.0516, 167.0790, 137.0500, 135.0440, 121.1015, 107.0480
8	20.88	5-methoxypodophyllotoxin	C_23_H_24_O_9_	445.1478		117.0695
9	20.94	ethyl p-methoxycinnamate	C_12_H_14_O_3_	207.1015		161.0595, 133.0641, 103.0537
10	27.42	quercetin 3-(6-O-acetyl-beta-glucoside)	C_23_H_22_O_13_	507.1158		105.0332, 117.0698
11	1.22	p-hydroxybenzoic acid	C_7_H_6_O_3_		137.0244	137.0226, 108.0207
12	1.76	Methyl 3,4-dihydroxybenzoate	C_8_H_8_O_4_		167.0349	107.0506
13	2.4	vanillic acid	C_8_H_8_O_4_		167.0349	123.0420
14	3.77	phenylmethanol	C_7_H_8_O		107.0502	107.0479, 106.0404
15	4.39	p-hydroxycinnamic acid	C_9_H_8_O_3_		163.0400	119.0497, 117.0345
16	4.51	1-O-4-Carboxylphenyl-(6-O-4-hydroxybenzoyl)-β- D-glucopyranoside	C_20_H_20_O_10_		419.0983	281.0646, 137.0235
17	4.72	ferulic acid	C_10_H_10_O_4_		193.0506	134.0359, 133.0281, 132.0196, 117.0331, 106.0411
18	4.96	benzoic acid	C_7_H_6_O_2_		121.0295	121.0288, 120.0209, 108.0207
19	5.25	hedycoropyran B	C_20_H_24_O_7_		375.1449	177.0548, 163.0393, 135.0442
20	5.29	kaempsulfonic acid A	C_20_H_24_O_8_S		423.1119	423.1984, 423.1706, 287.0566, 267.1585, 243.0304, 229.1095, 135.0441
21	5.29	kaempsulfonic acid B	C_20_H_24_O_8_S		423.1119	423.1984, 423.1706, 287.0566, 267.1585, 243.0304, 229.1095, 135.0441
22	5.64	(3R,5S)-3,5-dihydroxy-1,7-bis(3,4-dihydroxyphenyl) heptane	C_19_H_24_O_6_		347.1500	347.1477, 165.0547, 163.0757, 137.0600
23	6.11	(1R,3R,5R)-1,5-epoxy-3-hydroxy-1-(3,4-dihydroxyphenyl)-7-(3,4-dihydroxyphenyl) heptane	C_19_H_22_O_6_		345.1343	209.0814, 165.0549, 161.0600, 135.0440
24	6.21	p-methoxybenzoic acid	C_8_H_8_O_3_		151.0400	150.2345, 122.0327, 108.0205
25	6.67	(1R,2S,4R)-p-menth-5-ene-1,2,8-triol	C_10_H_18_O_3_		185.1183	139.1110
26	7.72	phaeoheptanoxide	C_19_H_22_O_5_		329.1394	161.0601, 159.0443, 134.0368, 135.0438
27	8.33	p-methoxycinnamic acid	C_10_H_10_O_3_		177.0557	161.0592, 119.0453, 117.0336
28	8.86	ethyl cinnamate	C_11_H_12_O_2_		175.0764	159.0443, 131.0478
29	9.76	(3R,4R,6S)-3,6-dihydroxy-1-menthene	C_10_H_18_O_2_		169.1234	125.0235, 107.0129
30	12.18	4-methoxy-benzyl (E)-3-(4-methoxyp-henyl) acrylate	C_18_H_18_O_4_		297.1132	253.1220, 235.1648, 121.0434, 107.0487
31	19.55	kaempferide	C_16_H_12_O_6_		299.0561	299.0527, 271.0593, 271.0831, 263.2009, 257.0372, 163.0058
32	26.71	kaempferol	C_15_H_10_O_6_		285.0404	285.0390, 257.0428, 217.0507, 147.0777
33	26.75	luteolin	C_15_H_10_O_6_		285.0404	285.0377, 271.1278, 257.0431, 241.0483, 201.0543, 159.0404, 157.0656
34	30.53	dibutyl phthalate	C_16_H_22_O_4_		277.1445	277.1423, 233.1539, 217.1216,
35	31.22	monopalmitin	C_19_H_38_O_4_		329.2697	329.2645, 329.2264, 257.1844
36	31.42	sandaracopimaradien-6β,9α-diol-l-one	C_20_H_30_O_3_		317.2122	273.2196, 271.2070, 149.0980
37	32.22	kaemgalangol A	C_20_H_30_O_3_		317.2122	273.2196
38	34.44	linolenic acid	C_18_H_30_O_2_		277.2173	277.2157, 147.0781
39	36.59	6β-acetoxysandaracopimaradiene-1α,9α-diol	C_22_H_34_O_4_		361.2384	269.1882, 215.1805
40	39.69	6β-acetoxy-1α-14α-dihydroxyisopimara-8(9),15-diene	C_22_H_34_O_4_		361.2384	283.2615, 269.1875
41	39.47	stearic acid	C_18_H_36_O_2_		283.2642	283.2621, 265.2518
42	43.22	6β-hydroxypimara-8(14),15-diene-1-one	C_20_H_30_O_2_		301.2173	301.2138, 255.2591
43	44.00	linoleic acid	C_18_H_32_O_2_		279.2329	279.2314, 261.2201

**Table 2:** Radical scavenging activity of KGEA.

Samples	DPPH (EC_50_)	ABTS (IC_50_)
KGEA (mg/mL)	4.88 ± 0.74	1.07 ± 0.06
Ascorbic acid (μg/mL)	22.64 ± 0.85	14.83 ± 0.74

## Data Availability

Data are contained within the article and Supplementary Materials.

## Supplementary Materials

The following supporting information can be downloaded at: https://www.mdpi.com/article/10.3390/antiox14050551/s1, Figure S1: UPLC-MS analysis of KGEA.

## Abbreviations

The following abbreviations are used in this manuscript:

KG	*Kaempferia galanga* L.
KGEE	the ethanolic extract from *K. galanga*
KGPE	the petroleum ether fraction of ethanolic extract from *K. galanga*
KGEA	the ethyl acetate fraction of ethanolic extract from *K. galanga*
KGNB	the n-butanol fraction of ethanolic extract from *K. galanga*
KGAS	the remaining aqueous solution fraction of ethanolic extract from *K. galanga*
UV	ultraviolet
ROS	reactive oxygen species
TCM	traditional Chinese medicine
MD	molecular dynamics
H_2_O_2_	hydrogen peroxide
DPPH	2,2-diphenyl-1-picrylhydrazyl
ABTS	2,2′-amino-di(2-ethyl benzothiazoline sulfonic acid-6) ammonium salt
DCF-DA	2′,7′-dichlorodihydrofluorescein diacetate
DPPP	diphenyl-1-pyrenylphosphine
MDA	malondialdehyde
SOD	superoxide dismutase
CAT	catalase
GSH-Px	glutathione peroxidase
UHPLC-QTOF-MS	ultra-high-performance liquid chromatography-quadrupole time-of-flight mass spectrometry
SMILES	simplified molecular input line entry system
ADME	absorption, distribution, metabolism, excretion
PPI	protein–protein interactions
GO	gene ontology
KEGG	Kyoto encyclopedia of genes and genomes
RMSD	root-mean-square deviation
RMSF	root mean square fluctuation
Rg	radius of gyration
RMSF	root mean square fluctuation
ECL	enhanced chemiluminescence
AKT	protein kinase B
p-AKT	phospho protein kinase B
PI3K	phosphoinositide 3-kinase
p-PI3K	phospho phosphoinositide 3-kinase
ERK1/2	extracellular signal-regulated kinase 1/2
p-ERK1/2	phospho extracellular signal regulated kinase 1/2
CCK-8	cell counting kit-8 (2-(2-methoxy-4-nitrophenyl)-3-(4-nitrophenyl)-5-(2,4-disulfophenyl)-2H-tetrazolium, monosodium salt)
JC-1	5,5′,6,6′-tetrachloro-1,1′,3,3′-tetraethyl-benzimidazolylcarbocyanine Chloride

## References

[1] Ungvari Z., Bagi Z., Feher A., Recchia F.A., Sonntag W.E., Pearson K., Cabo R.D., Csiszar A. (2010). Resveratrol confers endothelial protection via activation of the antioxidant transcription factor Nrf2. Am. J. Physiol. Heart. Circ..

[2] Sahreen S., Khan M.R., Khan R.A. (2010). Evaluation of antioxidant activities of various solvent extracts of Carissa opaca fruits. Food Chem..

[3] Wu W.L., Papagiannakopoulos T. (2020). The pleiotropic role of the Keap1/Nrf2 pathway in cancer. Annu. Rev. Cancer Biol..

[4] Zhu Q., Zeng J., Li J., Chen X.M., Miao J.X., Jin Q.Y., Chen H.Y. (2020). Effects of compound Centella on oxidative stress and Keap1-Nrf2-ARE pathway expression in diabetic kidney disease rats. Evid.-Based Compl. Alt..

[5] Guo Z., Mo Z. (2020). Keap1-Nrf2 signaling pathway in angiogenesis and vascular diseases. J. Tissue Eng. Regener. Med..

[6] Aynur S., Bircan C.T., Sevil E.T., Sevcan A., Göksel K., Süleyman K., Murat K. (2016). Assessment of the Antioxidant Activity of *Silybum marianum* Seed Extract and Its Protective Efect Against DNA Oxidation, Protein Damage and Lipid Peroxidation. Food Technol. Biotechnol..

[7] Ghosh T., Basu A., Adhikari D., Roy D., Pal A.K. (2015). Antioxidant activity and structural features of *Cinnamomum zeylanicum*. 3 Biotech.

[8] Munekata P.E.S., Gullon B., Pateiro M., Tomasevic I., Domínguez R., Lorenzo J.M. (2020). Natural Antioxidants from Seeds and Their Application in Meat Products. Antioxidants.

[9] Li P., Feng B., Jiang H., Han X., Wu Z.F., Wang Y.Q., Lin J.Z., Zhang Y., Yang M., Han L. (2018). A Novel Forming Method of Traditional Chinese Medicine Dispersible Tablets to Achieve Rapid Disintegration Based on the Powder Modifcation Principle. Sci. Rep..

[10] Wang S.Y., Zhao H., Xu H.T., Han X.D., Wu Y.S., Xu F.F., Yang X.B., Göransson U., Liu B. (2021). *Kaempferia galanga* L.: Progresses in phytochemistry, pharmacology, toxicology and ethnomedicinal uses. Front. Pharmacol..

[11] Munda S., Saikia P., Lal M. (2018). Chemical composition and biological activity of essential oil of *Kaempferia galanga*: A review. J. Essent. Oil Res..

[12] Mustafa R.A., Hamid A.A., Mohamed S., Bakar F.A. (2010). Total phenolic compounds, flavonoids, and radical scavenging activity of 21 selected tropical plants. J. Food Sci..

[13] Jantan I.B., Yassin M.S.M., Chin C.B., Chen L.L., Sim N.L. (2003). Antifungal activity of the essential oils of nine zingiberaceae species. Pharm. Biol..

[14] Sulaiman M.R., Zakaria Z.A., Daud I.A., Ng F.N., Ng Y.C., Hidayat M.T. (2008). Antinociceptive and anti-inflammatory activities of the aqueous extract of *Kaempferia galanga* leaves in animal models. J. Nat. Med..

[15] Ali H., Yesmin R., Satter A., Habib R., Yeasmin T. (2018). Antioxidant and anti-neoplastic activities of methanolic extract of *Kaempferia galanga* linn. Rhizome against Ehrlich ascites carcinoma cells. J. King Saud Univ. Sci..

[16] Rahman I., Kabir T., Islam N., Muqaddim M., Sharmin S., Ullah M.S., Uddin S. (2019). Investigation of Antioxidant and Cytotoxic Activities of *Kaempferia galanga* L. Res. J. Pharm. Technol..

[17] Hopkins A.L. (2008). Network pharmacology: The next paradigm in drug discovery. Nat. Chem. Biol..

[18] Law V., Knox C., Djoumbou Y., Jewison T., Guo A.C., Liu Y.F., Maciejewski A., Arndt D., Wilson M., Neveu V. (2014). DrugBank 40: Shedding new light on drug metabolism. Nucleic Acids Res..

[19] Zhang R., Zhu X., Bai H., Ning K. (2019). Network pharmacology databases for traditional Chinese medicine: Review and assessment. Front. Pharmacol..

[20] Babgi B.A., Alsayari J., Alenezi H.M., Abdellatif M.H., Eltayeb N.E., Emwas A.H.M., Jaremko M., Hussien M.A. (2021). Alteration of Anticancer and Protein-Binding Properties of Gold(I) Alkynyl by Phenolic Schif Bases Moieties. Pharmaceutics.

[21] Mu C., Sheng Y., Wang Q., Wang Q., Amin A., Li X.G., Xie Y.Q. (2021). Potential compound from herbal food of *Rhizoma polygonati* for treatment of COVID-19 analyzed by network pharmacology: Viral and cancer signaling mechanisms. J. Funct. Foods.

[22] Zhou W., Chen Z., Li W., Wang Y., Li X., Yu H., Ran P., Liu Z. (2019). Systems pharmacology uncovers the mechanisms of anti-asthma herbal medicine intervention (ASHMI) for the prevention of asthma. J. Funct. Foods.

[23] Nie J.Y., Li R., Jiang Z.T., Wang Y., Tan J., Tang S.H., Zhang Y. (2020). Screening and evaluation ofradical scavenging active compounds in the essential oil from *Magnolia biondii* pamp byelectronic nose coupled with chemical methodology. Ind. Crops Prod..

[24] Tungcharoen P., Wattanapiromsakul C., Tansakul P., Nakamura S., Matsuda H., Tewtrakul S. (2020). Anti-inflammatory Effect of Isopimarane Diterpenoids from *Kaempferia galanga*. Phytother. Res..

[25] Yao F., Huang Y., Wang Y., He X. (2018). Anti-inflammatory Diarylheptanoids and Phenolics from the Rhizomes of Kencur (*Kaempferia galanga* L.). Ind. Crops Prod..

[26] Yao F.Z. (2018). Study on the Chemical Constituents of the Rhizome of *Kaempferia galanga* L. Master’s Thesis.

[27] Jiao Z.X., Xu W.F., Zheng J.S., Shen P., Qin A., Zhang S.Y., Yang C. (2017). Kaempferide Prevents Titanium Particle Induced Osteolysis by Suppressing JNK Activation During Osteoclast Formation. Sci. Rep..

[28] Wu H.D. (2016). Study on the Chemical Constituents of Rhizoma Kaempferiae. Master’s Thesis.

[29] Piao C.L., Zhang Q., Jin D., Wang L., Zhang N.W., Lian F.M., Tong X. (2020). A Study on the Mechanism of Milkvetch Root in the Treatment of Diabetic Nephropathy Based on Network Pharmacology. Evid.-Based Complement. Altern. Med..

[30] Lobanov M., Bogatyreva N.S., Galzitskaia O.V. (2008). Radius of gyration is indicator of compactness of protein structure. Mol. Biol..

[31] García-Sánchez A., Miranda-Díaz A.G., Cardona-Muñoz E.G. (2020). The Role of Oxidative Stress in Physiopathology and Pharmacological Treatment with Pro- and Antioxidant Properties in Chronic Diseases. Oxid. Med. Cell. Longev..

[32] Liang B., Zhu Y.C., Lu J., Gu N. (2021). Effects of Traditional Chinese Medication-Based Bioactive Compounds on Cellular and Molecular Mechanisms of Oxidative Stress. Oxid. Med. Cell. Longev..

[33] Maestri D.M., Nepote V., Lamarque A.L., Zygadlo J.A. (2006). Natural products as antioxidants. Adv. Res..

[34] Ismail B.B., Pu Y.F., Guo M.M., Ma X.B., Liu D.H. (2019). LC-MS/QTOF identification of phytochemicals and the effects of solvents on phenolic constituents and antioxidant activity of baobab (*Adansonia digitata*) fruit pulp. Food Chem..

[35] Zhang Y., Wang G., Wang T., Cao W., Zhang L.X., Chen X.Y. (2019). Nrf2-Keap1 pathway-mediated effects of resveratrol on oxidative stress and apoptosis in hydrogen peroxide-treated rheumatoid arthritis fibroblast-like synoviocytes. Ann. N. Y. Acad. Sci..

[36] Qiang Z., Pan J.K., Liu H., Jiao Z.G. (2023). Characterization of the Synergistic Antioxidant Activity of Epigallocatechin Gallate (EGCG) and Kaempferol. Molecules.

[37] Liu W., Yin D.X., Zhang T., Hou X., Qiao Q., Song P. (2020). Major Fatty Acid Compositions and Antioxidant Activity of Cultivated Paeonia ostii under Different Nitrogen Fertilizer Application. Chem. Biodivers..

[38] Zhang J.F., Yang Y.X., Han H.L., Zhang L.L., Wang T. (2021). Bisdemethoxycurcumin Protects Small Intestine from Lipopolysaccharide-Induced Mitochondrial Dysfunction via Activating Mitochondrial Antioxidant Systems and Mitochondrial Biogenesis in Broiler Chickens. Oxid. Med. Cell. Longev..

[39] Shabani M., Jamali Z., Bayrami D., Salimi A. (2023). Vanillic acid alleviates methamphetamine-induced mitochondrial toxicity in cardiac mitochondria via antioxidant activity and inhibition of MPT Pore opening: An in-vitro study. BMC Pharmacol. Toxicol..

[40] Boeing T., Souza P.D., Speca S., Somensi L.B., Mariano L.N.B., Cury B.J., Anjos M.F.D., Quintão N.L.M., Dubuqoy L., Desreumax P. (2020). Luteolin prevents irinotecan-induced intestinal mucositis in mice through antioxidant and anti-inflammatory properties. Br. J. Pharmacol..

[41] Ma X.L., Tian Y., Xue K.Y., Huai Y., Patil S.Y., Deng X.N., Hao Q., Li D.M., Miao Z.P., Zhang W.J. (2021). Kaempferide enhances antioxidant capacity to promote osteogenesis through FoxO1/β-catenin signaling pathway. Eur. J. Pharmacol..

[42] Imran M., Rauf A., Shah Z.A., Saeed F., Imran A., Arshad M.U., Ahmad B., Bawazeer S., Atif M., Peters D.G. (2019). Chemo-preventive and therapeutic effect of the dietary flavonoid kaempferol: A comprehensive review. Phytother. Res..

[43] Akihiro T., Atsuko I., Hideyuki I. (2017). Structural evidence for the DPPH radical-scavenging mechanism of 2-Oa-D-glucopyranosyl-L ascorbic acid. Bioorg. Med. Chem..

[44] Zhen J., Villani T.S., Guo Y., Qi Y., Chin K., Pan M.H., Wu Q. (2016). Phytochemistry, antioxidant capacity, total phenolic content and anti-inflammatory activity of Hibiscus sabdariffa leaves. Food Chem..

[45] Yang S., Lian G. (2020). ROS and diseases: Role in metabolism and energy supply. Mol. Cell. Biochem..

[46] Vahdati S., Lashkari A., Navasatli A.S., Ardestani K.S. (2020). and M. Safavi. Butylated hydroxyl-toluene, 2,4-Di-tert-butylphenol, and phytol of Chlorella sp. protect the PC12 cell line against H_2_O_2_-induced neurotoxicity. Biomed. Pharmacother..

[47] Wen L., Zheng G., You L., Abbasi A.M., Li T., Fu X., Liu R.H. (2016). Phytochemical profiles and cellular antioxidant activity of Malus doumeri (bois) chevalier on 2,2′-azobis (2-amidinopropane) dihydrochloride (ABAP)-induced oxidative stress. J. Funct. Foods.

[48] Jiang J., Zhuang J.Y., Fan Y.Y., Shen B. (2009). Mapping of QTLs for leaf malondialdehyde content associated with stress tolerance in rice. Rice Sci..

[49] Pham-Huy L.A., He H., Pham-Huy C. (2008). Free radicals, antioxidants in disease and health. Int. J. Biomed. Sci..

