# Supplementary material for: Antioxidant Effect of Ethyl Acetate Fraction from Kaempferia galanga L.: Integrated Phytochemical Profiling, Network Analysis, and Experimental Validation
Source: Antioxidants (Basel). 2025 May 5;14(5):551. doi: 10.3390/antiox14050551 (PMC12108274; doi:10.3390/antiox14050551)
Supplement: Supplementary file 1 [file antioxidants-14-00551-s001.zip › antioxidants-3555829-supplementary.pdf]

Supplementary material

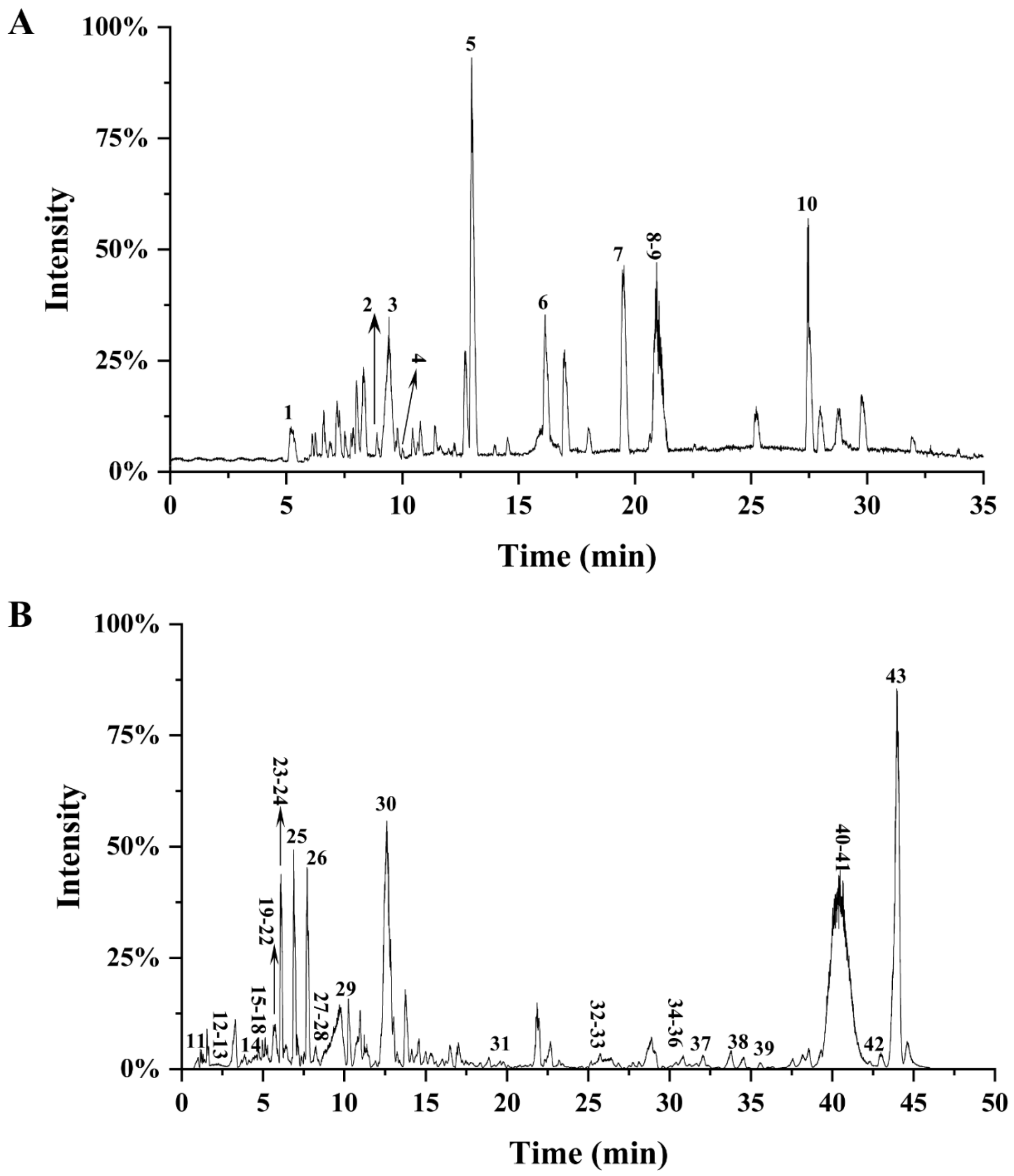

Figure S1. UHPLC-MS analysis of KGEA. (A) positive ion mode. (B) negative ion mode.
